# Mild electrical stimulation with heat shock attenuates renal pathology in adriamycin-induced nephrotic syndrome mouse model

**DOI:** 10.1038/s41598-020-75761-8

**Published:** 2020-10-30

**Authors:** Keisuke Teramoto, Yu Tsurekawa, Mary Ann Suico, Shota Kaseda, Kohei Omachi, Tsubasa Yokota, Misato Kamura, Mariam Piruzyan, Tatsuya Kondo, Tsuyoshi Shuto, Eiichi Araki, Hirofumi Kai

**Affiliations:** 1 Department of Molecular Medicine, Graduate School of Pharmaceutical Sciences, Kumamoto University, 5-1 Oe-honmachi, Chuo-ku, Kumamoto, Kumamoto 862-0973 Japan; 2 Program for Leading Graduate Schools “HIGO (Health Life Science: Interdisciplinary and Global Oriented) Program”, Kumamoto University, Kumamoto, Japan; 3 Global Center for Natural Resources Sciences, Faculty of Life Sciences, Kumamoto University, 5-1 Oe-Honmachi, Chuo-ku, Kumamoto, Kumamoto 862-0973 Japan; 4 Department of Metabolic Medicine, Faculty of Life Sciences, Kumamoto University, 1-1-1 Honjo, Chuo-ku, Kumamoto, Kumamoto 860-8556 Japan

**Keywords:** Alport syndrome, Molecular medicine

## Abstract

Nephrotic syndrome (NS) is a renal disorder that is characterized by massive proteinuria, hypoalbuminemia and edema. One of the main causes of NS is focal segmental glomerulosclerosis (FSGS), which has extremely poor prognosis. Although steroids and immunosuppressants are the first line of treatment, some FSGS cases are refractory, prompting the need to find new therapeutic strategies. We have previously demonstrated that an optimized combination treatment of mild electrical stimulation (MES) and heat shock (HS) has several biological benefits including the amelioration of the pathologies of the genetic renal disorder Alport syndrome. Here, we investigated the effect of MES + HS on adriamycin (ADR)-induced NS mouse model. MES + HS suppressed proteinuria and glomerulosclerosis induced by ADR. The expressions of pro-inflammatory cytokines and pro-fibrotic genes were also significantly downregulated by MES + HS. MES + HS decreased the expression level of cleaved caspase-3 and the number of TUNEL-positive cells, indicating that MES + HS exerted anti-apoptotic effect. Moreover, MES + HS activated the Akt signaling and induced the phosphorylation and inhibition of the apoptotic molecule BAD. In in vitro experiment, the Akt inhibitor abolished the MES + HS-induced Akt-BAD signaling and anti-apoptotic effect in ADR-treated cells. Collectively, our study suggested that MES + HS modulates ADR-induced pathologies and has renoprotective effect against ADR-induced NS via regulation of Akt-BAD axis.

## Introduction

Nephrotic syndrome (NS) is a renal disorder that is characterized by massive proteinuria, hypoalbuminemia and edema. Renal damage and glomerular injury cause NS and leakage of protein and albumin into the urine. The resulting hypoalbuminemia induces disruption of osmotic balance and low-density lipoprotein (LDL) production followed by edema and dyslipidemia^1^. As first line of treatment for NS, steroids are used, which is in fact effective for some types of NS. However, some NS patients exhibit severe phenotypes in which conventional medications do not work. The main histological findings in case of incurable NS, including non-response to steroids, are FSGS, minimal-change nephropathy and mesangial proliferative glomerulonephritis^2–4^. Particularly, FSGS has poor prognosis and often develops to end stage renal disease (ESRD)^5^. In addition to steroids, immunosuppressants are also used for NS therapy^6^, but neither agents can avoid causing side effects due to long-term medication. Furthermore, it is known that NS is caused by multiple factors such as cell damage, dysregulation of immune system, humoral factors and glomerular permeability factors^7–9^. The main pathomorphological consequences in NS are glomerulosclerosis and tubulointerstitial fibrosis. Fibrosis and acute inflammation that presage sclerosis have vital roles in the development and progression of proteinuric kidney pathologies in NS. These pathophysiological events are accompanied or triggered by apoptosis^10^. Programmed cell death or apoptosis is an active response to changes in the microenvironment and is characterized by the induction of specific intracellular cell death pathways^11^. The multifactorial pathogenesis mechanisms occurring in NS make it difficult to establish NS therapy. Because of these, development of novel NS therapy that is effective for FSGS is urgently needed.

It has been widely accepted that mechanical stimulation has various medical benefits including, but not limited to, muscular dystrophy recovery, nerve regeneration and wound healing^12–15^. Particularly, it has been shown that electrical stimulation influences cellular migration and wound healing via activating phosphatidylinositol 3-OH kinase (PI3K)-Akt pathway^16^. Our previous works on mild electrical stimulation (MES) with heat shock (HS) have revealed that this combination treatment is effective in several disease models^17–19^. Moreover, we have investigated the biological effects of optimized MES + HS treatment and elucidated some of the mechanistic pathways responsible for the effectiveness of MES + HS. Using murine models, we have previously shown that MES + HS ameliorated the pathologies in type 2 diabetes mellitus (T2DM), obesity, hepatic ischemia reperfusion injury, and skin psoriasis^17–19^. In clinical investigations, we have reported that MES + HS improved insulin resistance, glucose metabolism parameters and inflammatory parameters in T2DM patients without adverse effects, pointing to the safety of MES + HS^20–22^. Interestingly, MES + HS tended to reduce renal injury parameters such as blood urine nitrogen (BUN). Furthermore, we demonstrated previously that MES + HS suppressed renal inflammation in a mouse model of Alport syndrome, a hereditary kidney disease^23^. Collectively, MES + HS attenuates these disease conditions mostly by modulating injury, fibrosis and the immune response through suppression of cytokines and fibrotic factors via multiple signaling pathways such as the PI3K-Akt, mitogen-activated protein kinases (MAPKs) and transforming growth factor (TGF)-β, among others. Although there is accumulating lines of evidence on the beneficial biological effects of MES + HS, it is unclear whether MES + HS has a protective effect on renal cell damage or NS. In this study, we investigated the effects of MES + HS on NS using adriamycin (ADR; also called doxorubicin)-induced NS mouse model^24^. This model is also known to be accompanied by FSGS. MES + HS treatment ameliorated renal function disorders, histological abnormalities and inflammation in NS mice. Moreover, MES + HS suppressed the NS-induced apoptosis via regulating Akt-BAD axis, indicating the potential of MES + HS as a novel therapeutic modality for NS.

## Results

### MES + HS treatment ameliorates the renal dysfunction in ADR-induced NS mouse model

**Figure 1:**
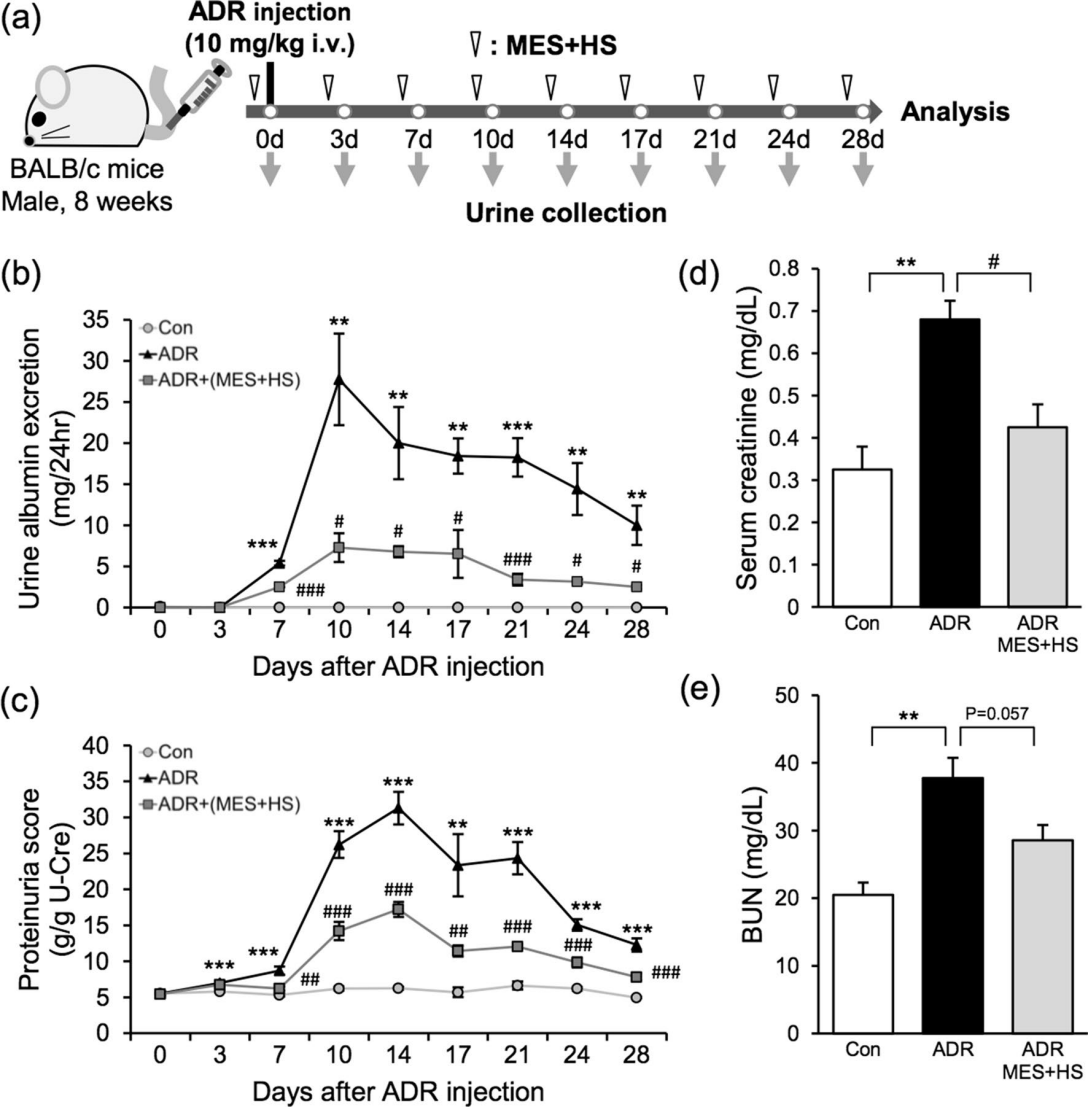
MES + HS ameliorates renal dysfunction in ADR-induced NS mice. (**a**) Experimental diagram originally drawn by us. Eight-week-old male mice were injected with ADR (10 mg/kg; i.v.) and treated with MES + HS for 10 min twice a week for 4 weeks. MES + HS treatment was started one day prior to injection with ADR. Urine samples were collected twice a week. (**b**) Urinary albumin excretion was assessed by 12% SDS-PAGE followed by CBB staining and densitometric analysis of gel blots. (**c**) Urinary protein and creatinine were assessed by Bradford’s and Jaffe’s method, respectively. Urinary protein concentration was normalized with urinary creatinine concentration. (**d**) Serum creatinine and (**e**) blood urea nitrogen (BUN) scores of murine plasma was measured using DRI-CHEM (Fuji). Values are the mean ± SE. n = 4–5 mice per group. **p < 0.01, ***p < 0.001 (Con vs ADR), ^#^p < 0.05, ^##^p < 0.01, ^###^p < 0.001 [ADR vs ADR + (MES + HS)] assessed by Tukey–Kramer method.

ADR-induced NS is an established murine model of developing nephrosis and FSGS caused by podocyte injury^24,25^. We used this model to evaluate the effect of MES + HS on NS. BALB/c mice were treated with MES + HS for 10 min twice a week starting one day prior to ADR injection (Fig. 1a). Albuminuria was notably elevated at 7 days, and peaked sharply at 10 days after ADR injection (black triangles; Fig. 1b). MES + HS treatment significantly decreased the albuminuria by 50% at 7 days, and caused a 75% reduction of albuminuria at 10 days after ADR injection (gray squares; Fig. 1b). The observed proteinuria was maximal at 10–14 days after ADR administration, but treatment with MES + HS reduced the ADR-induced proteinuria by 60–80% at these time points and thereafter (Fig. 1c). MES + HS treatment clearly and sustainably suppressed albuminuria and proteinuria. We also measured serum parameters for renal injury after ADR treatment. ADR increased the serum creatinine and BUN levels to approximately twofold when assessed at 28 days after ADR injection. These serum parameters of renal function were improved by MES + HS treatment (Fig. 1d,e). ADR-induced increase in serum creatinine and BUN were reduced by MES + HS with 37.5% and 24% reduction, respectively, compared with ADR-treated group. Collectively, these data suggested that MES + HS ameliorates renal dysfunction in mice induced by ADR.

### MES + HS treatment improves the renal pathology in ADR-induced NS mouse model

**Figure 2:**
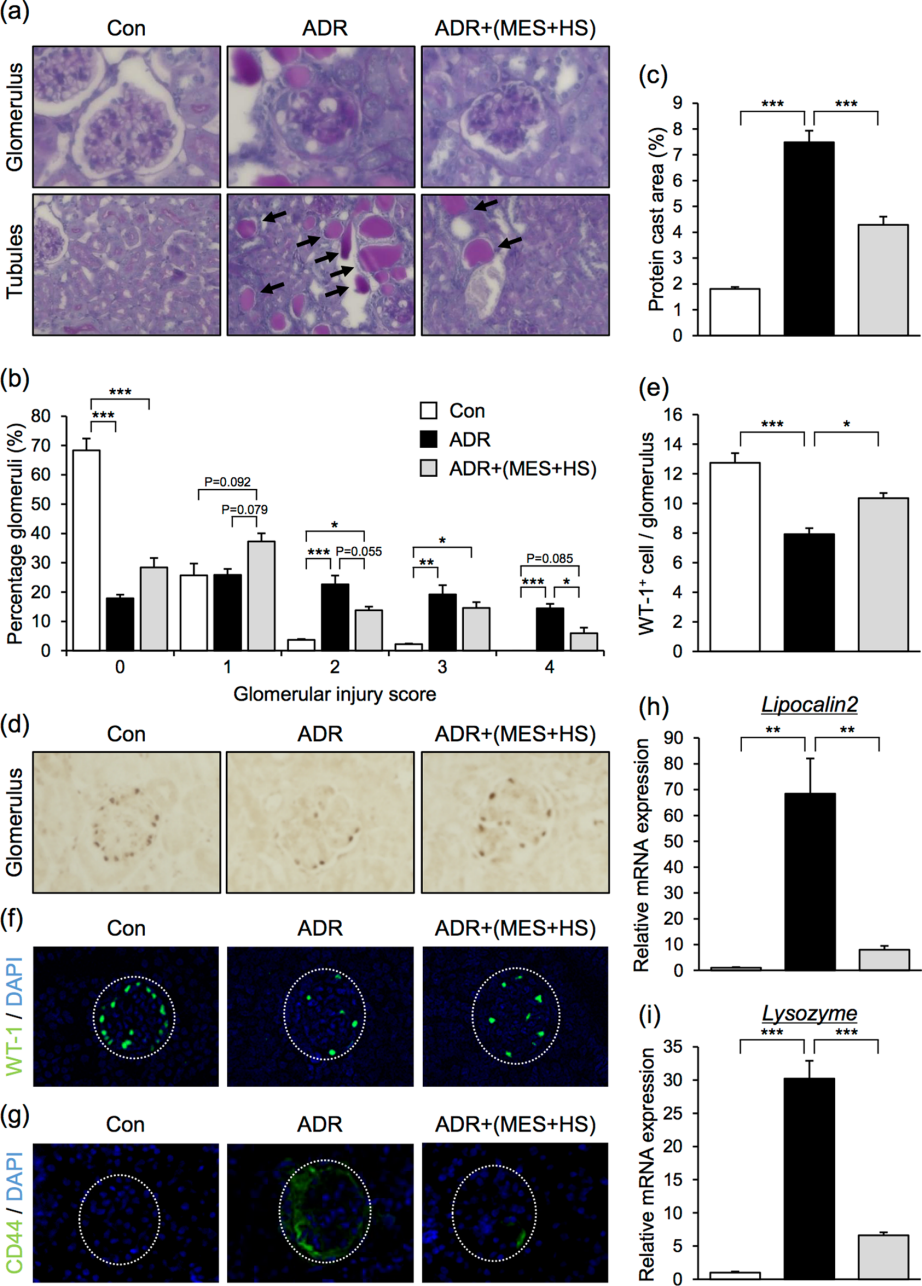
MES + HS improves the renal pathology in ADR-induced NS mouse model. (**a**) Images of PAS-stained kidney section. Upper images show glomerulus and lower images show tubules. Arrows indicate protein casts. (**b**,**c**) Bar graph shows quantification of glomerulosclerosis severity score and protein cast area, respectively. (**d**) Glomerular sections were immunostained to detect WT-1. (**e**) Bar graph shows the ratio of WT-1-positive cells to glomerulus. (**f**,**g**) Glomerular sections were stained with WT-1 and CD44, respectively, and visualized by immunofluorescence. (**h**,**i**) Total RNA was isolated from renal tissues and assessed by quantitative RT-PCR analysis. Bar graphs show gene expression levels of renal injury markers (*Lipocalin2*, *Lysozyme*). Values were normalized to 18S ribosomal RNA (internal control). Values are the mean ± SE. n = 4–5 mice per group. *p < 0.05, **p < 0.01, ***p < 0.001 assessed by Tukey–Kramer method.

It is known that ADR causes glomerular injury followed by glomerulosclerosis^26^. To evaluate whether MES + HS treatment can affect the pathology in ADR-induced NS, we stained renal sections with PAS. As expected, impaired or sclerotic glomeruli with severity score of 2 to 4 were increased by ADR injection compared with control group (see “Methods” for assessment; Fig. 2a,b). Crescent formation and glomerular collapse were observed in the renal sections of ADR-treated mice with accompanying disappearance of the Bowman’s capsule (Fig. 2a, upper panel, ADR). The focal glomerular sclerosis seen in these mice is consistent with ADR administration^27^. Interestingly, MES + HS treatment ameliorated the glomerulopathy and decreased the number of severely sclerotic glomeruli (Fig. 2a (upper panels),b). Furthermore, MES + HS inhibited the formation of protein casts that were seen in the tubules of ADR-treated mice which indicated tubular injury (Fig. 2a (lower panels),c). MES + HS reduced the severely glomerulosclerotic lesions (severity score of 4) and protein casts by 59% and 43%, respectively, compared with ADR-treated mice (Fig. 2b,c). We also stained for WT-1, which is a podocyte marker, to investigate the effect of MES + HS on podocyte injury. The number of WT-1-positive cells per glomerulus was reduced by ADR injection, suggesting podocyte loss, but MES + HS treatment suppressed this decrease (Fig. 2d,e). Immunofluorescence analysis similarly revealed the reduction of WT-1-positive cells upon ADR treatment that was improved by MES + HS (Fig. 2f). In addition, we examined the expression of CD44, which is a marker for activated parietal epithelial cells (PECs). The presence of activated PECs has been associated with evolving FSGS^28^. CD44 expression was detected in the severely impaired glomeruli of ADR-treated mice. Treatment with MES + HS suppressed the ADR-induced CD44 expression (Fig. 2g). We also evaluated the expression of *Lipocalin2* and *Lysozyme*, which are markers of renal injury. The expression levels of these genes were remarkably upregulated by ADR treatment as revealed by quantitative RT-PCR analysis. But the ADR-induced expression of these genes was significantly reduced by MES + HS with 88% reduction for *Lipocalin2* and 78% reduction for *Lysozyme* (Fig. 2h,i). Taken together, MES + HS treatment limits renal injury and pathology induced by ADR.

### MES + HS suppresses the expression levels of pro-inflammatory cytokines and pro-fibrotic genes

**Figure 3:**
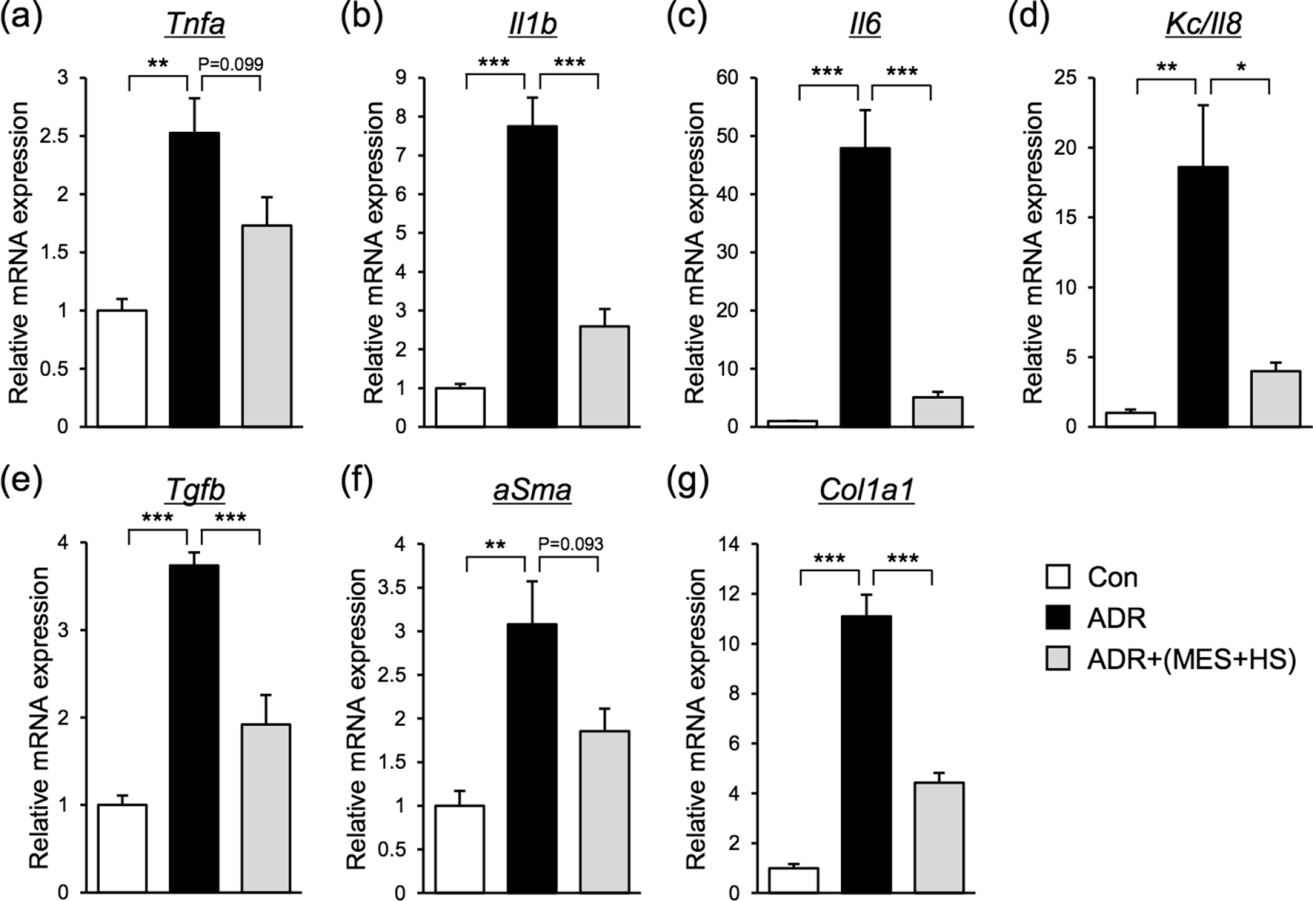
MES + HS suppresses the expression levels of pro-inflammatory cytokines and pro-fibrotic genes. (**a**–**g**) Total RNA was isolated from renal tissues, and pro-inflammatory (**a**–**d**) and pro-fibrotic genes (**e**–**g**) were assessed by quantitative RT-PCR. Values were normalized to 18S ribosomal RNA (internal control). Values are the mean ± SE. n = 4–5 mice per group. *p < 0.05, **p < 0.01, ***p < 0.001 assessed by Tukey–Kramer method.

Renal inflammation and fibrosis are key features associated with NS^7,29^. Therefore, we evaluated the effect of MES + HS treatment on NS-related inflammation by analyzing the mRNA expression levels of pro-inflammatory cytokines interleukin-1β (*Il1b*), *Il6*, *Kc/Il8* and tumor necrosis factor*-*α (*Tnfa*), and pro-fibrotic genes such as α-smooth muscle actin (α-*Sma*), *Tgfb* and collagen type 1 α1 chain (*Col1a1*). Consistent with the known effect of ADR on inflammation and fibrosis, the expression levels of the above-mentioned genes were remarkably increased by ADR administration. Depending on the gene, the upregulation was more than 2–40 times higher in ADR-treated mice than in control (Fig. 3). Notably, treatment with MES + HS reduced the elevated levels of proinflammatory cytokines *Tnfa*, *Il1b*, *Il6*, *Kc/Il8* by 31%, 66%, 89% and 79%, respectively (Fig. 3a–d), and profibrotic genes *Tgfb*, *aSma and Col1a1* by 49%, 42% and 60%, respectively (Fig. 3e–g). These results suggested that anti-inflammation and anti-fibrotic effects are part of the renoprotective effect of MES + HS treatment. We observed that *Tnfa* and *aSma* were only modestly induced by ADR at 2.5- and 3-fold, respectively. The reduction of these genes by MES + HS treatment was noticeable but not statistically significant (Fig. 3a,f). This suggested that the more dysregulated genes by ADR, such as *Il-6* and *Kc/Il8*, were more efficiently targeted by MES + HS.

### MES + HS treatment suppresses renal apoptosis in ADR-induced NS mice by activating the Akt pathway

**Figure 4:**
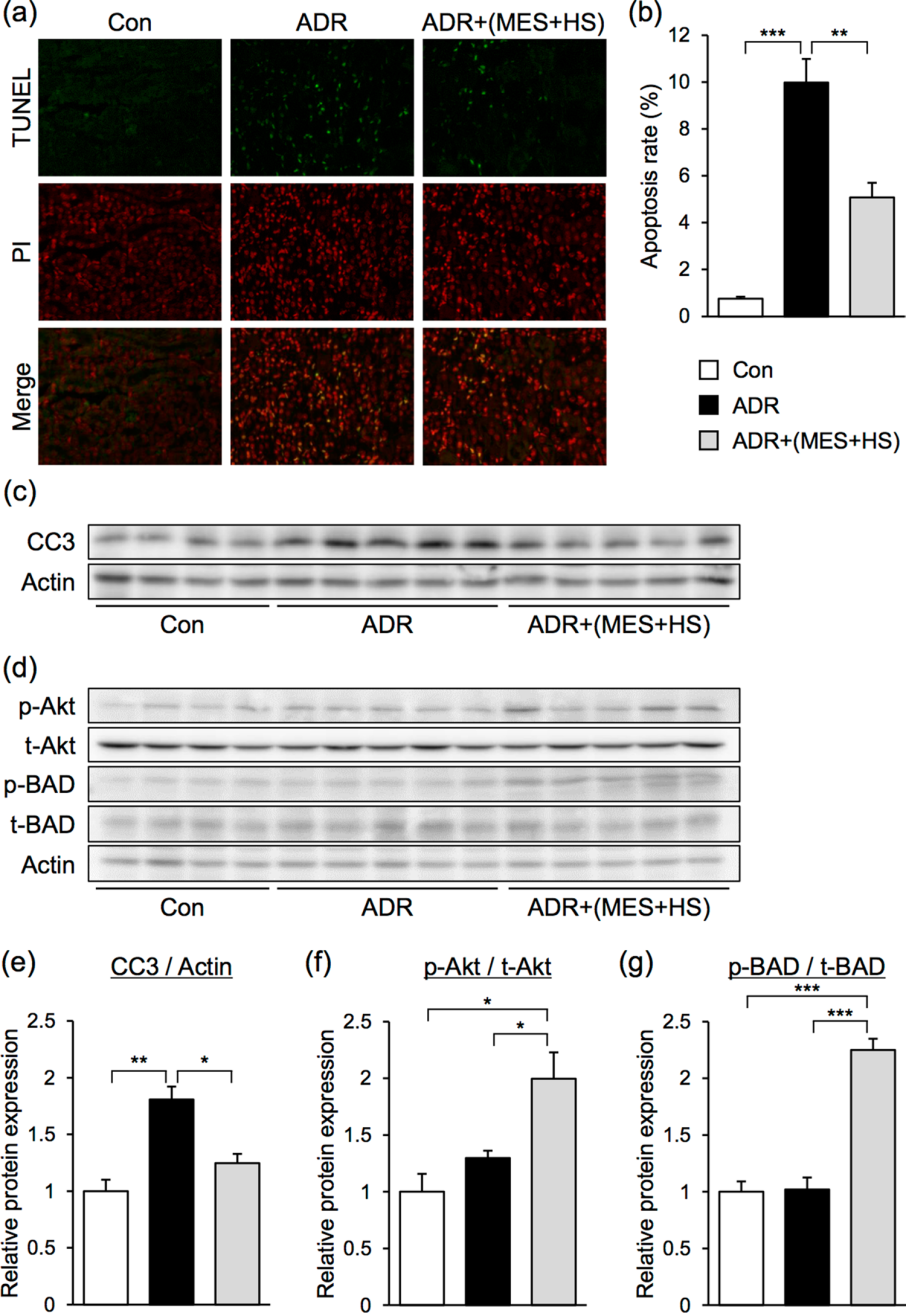
MES + HS suppresses apoptosis in ADR-induced NS mice. (**a**) Renal sections were stained with TUNEL for apoptotic cells. All cells were counterstained with PI. (**b**) Apoptosis rate was calculated by the ratio of TUNEL-positive cells to PI-positive cells. (**c**,**d**) Immunoblots of lysates isolated from mouse renal tissues were analyzed by western blotting using the indicated antibodies. Actin was used as loading control. Full-length blots are presented in Supplementary Figure 1. (**e**–**g**) Bar graph shows quantification of blots in (**c**,**d**). Expression of phosphorylated proteins were normalized with total protein expression. Graphs are the mean ± SE. n = 4–5 mice per group. *p < 0.05, **p < 0.01, ***p < 0.001 assessed by Tukey–Kramer method.

As an anti-cancer agent, ADR is known to induce cellular apoptosis. The ability of ADR to cause apoptosis is an important mechanism for its nephrotoxicity^30^. To evaluate whether MES + HS has anti-apoptotic effect in ADR-induced NS mice, we measured the number of renal apoptotic cells using TUNEL staining system. As expected, the number of TUNEL-positive cells were robustly increased in mouse kidney tissue by ADR administration (Fig. 4a,b). Interestingly, the number of these cells was reduced by MES + HS treatment by 50%, indicating that MES + HS treatment could suppress cell apoptosis. To confirm this result, we checked the expression levels of apoptosis-related proteins in kidney tissue samples. The protein level of cleaved caspase 3 (CC3), which is a marker of cellular apoptosis, was upregulated in renal tissues of ADR-treated mice, demonstrating that ADR caused renal apoptosis. The upregulated CC3 expression by ADR was significantly reduced (33% reduction) by treatment with MES + HS (Fig. 4c,e).

To examine the mechanistic pathway affected by MES + HS in ADR-induced apoptosis, we focused on the Akt pathway, which is one of the major anti-apoptotic pathways. One mechanism for the anti-apoptotic function of Akt involves the phosphorylation and inhibition of pro-apoptotic factor Bcl-xL/Bcl-2-associated death (BAD)^31^. First, we assessed the level of activated Akt. The expression of phosphorylated Akt (p-Akt) was increased in kidneys of MES + HS-treated mice group compared with control and ADR only-treated groups. The level of total Akt was not changed, indicating that MES + HS activated the Akt rather than increasing its basal expression (Fig. 4d,f). Furthermore, MES + HS treatment significantly increased the expression of phosphorylated BAD in NS mice kidneys (Fig. 4d,g). The phosphorylation of BAD by Akt is known to inhibit apoptosis^32^. Together, these results indicated that MES + HS treatment activates Akt signaling in vivo leading to the phosphorylation and inactivation of BAD and subsequent reduction of apoptosis.

**Figure 5:**
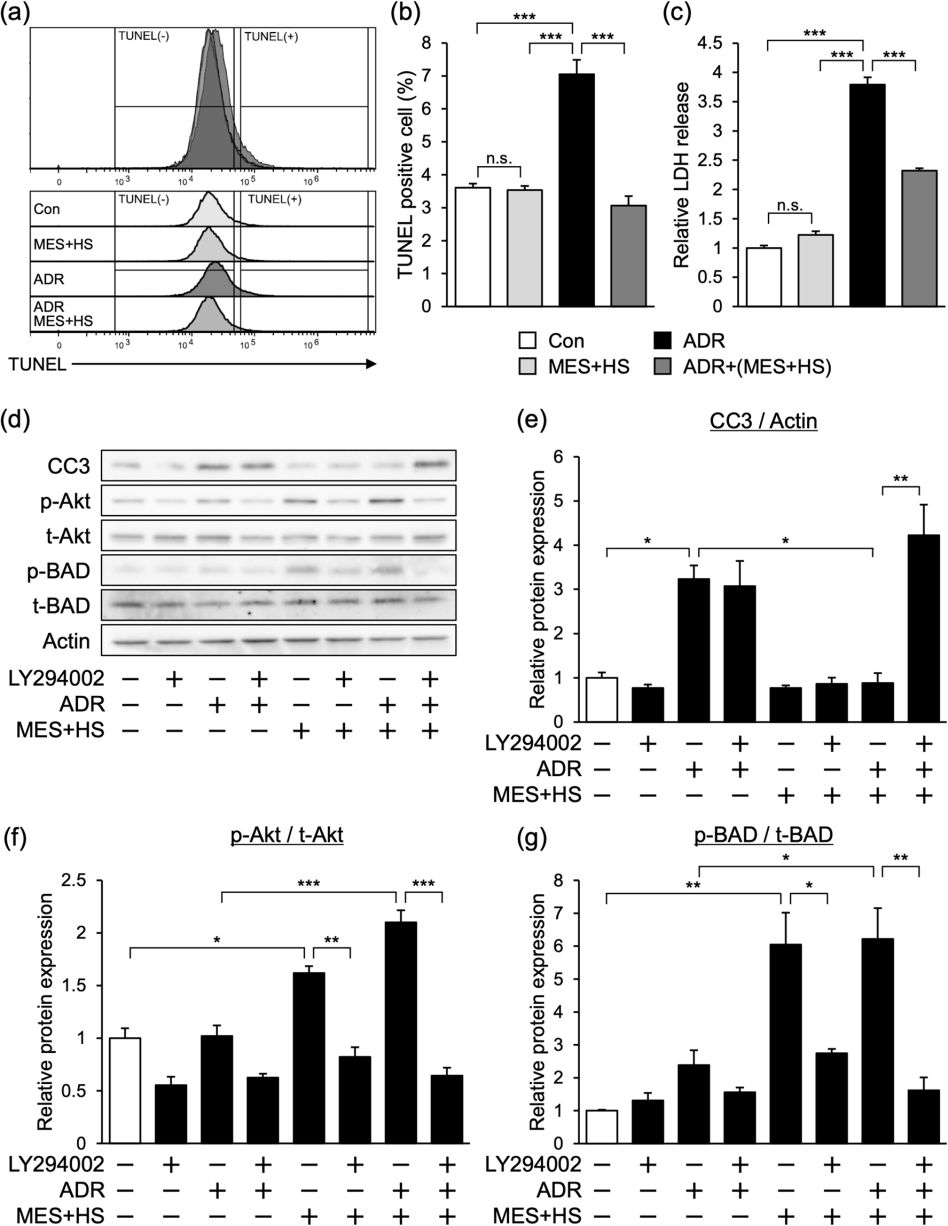
MES + HS suppresses ADR-induced apoptosis in MPC5 cell line. (**a**) MPC5 cells were treated with MES + HS (1 V/cm, 0.1 ms, 55 pps) for 10 min, and subsequently treated with 200 nmol/L ADR. Twenty-four hours after, cells were stained with TUNEL and analyzed by flow cytometry. (**b**) Data in (**a**) was statistically calculated and analyzed. (**c**) Relative LDH release was measured by colorimetric method in MPC cells treated with MES + HS and ADR. Released LDH concentration was normalized with intracellular LDH concentration. (**d**) MPC5 cells were treated with Akt phosphorylation inhibitor (LY294002; 10 µM) for 1 h before MES + HS and ADR (200 nmol/L) treatment. Protein lysates were collected 24 h after ADR treatment and analyzed by western blotting. Actin was used as loading control. Full-length blots are presented in Supplementary Figure 2. (**e**–**g**) Bar graph shows quantification of blots in (**d**). Expression of phosphorylated proteins were normalized with total protein expression. Values are the mean ± SE. n = 3 per group. *p < 0.05, **p < 0.01, ***p < 0.001 assessed by Tukey–Kramer method.

To verify these results, we used the mouse podocyte cell line MPC5. Cells were treated with 200 nmol/L ADR, and apoptotic cells were analyzed by flow cytometry using the fluorometric TUNEL system. ADR treatment induced a two-fold increase in TUNEL-positive cells. Notably, treatment with MES + HS significantly inhibited the ADR-induced apoptosis, such that the number of TUNEL-positive cells was similar to the control group (Fig. 5a,b). In ADR-treated cells, there was more than 3.5-fold increase in LDH release into the media, which is a marker of cellular damage. MES + HS significantly suppressed the ADR-induced release of LDH, by 40% (Fig. 5c). To determine the impact of MES + HS on apoptotic molecules, we checked the expression level of apoptosis-related proteins. Consistent with the in vivo results, the expression of CC3 was elevated by ADR treatment with threefold increase, but MES + HS suppressed this upregulation down to control level (Fig. 5d,e). We also observed that MES + HS increased the Akt activity with or without ADR by 1.6- to 2-fold (Fig. 5d,f). The phosphorylation of BAD, the downstream target of Akt to inhibit apoptosis, was increased by MES + HS treatment with or without ADR (Fig. 5d,g), which corresponds to the increase of activated Akt. To verify the involvement of Akt signaling in MES + HS-mediated inhibition of apoptosis, we treated the cells with Akt inhibitor LY294002. LY294002 efficiently suppressed the activation of Akt pathway by MES + HS treatment in MPC5 cells (Fig. 5d,f). Importantly, the blockade of Akt signaling pathway by LY294002 reversed the MES + HS-induced suppression of CC3, leading to the fourfold increase of CC3 expression level (Fig. 5d,e). Moreover, LY294002 treatment also inhibited the MES + HS-induced phosphorylated BAD expression in ADR-treated cells (Fig. 5d,g). These observations indicate that MES + HS suppresses ADR-induced cell death via the Akt-BAD axis. In conclusion, MES + HS exerts renoprotective effect against ADR by limiting renal inflammation and fibrosis and reducing renal cell apoptosis.

## Discussion

In ADR nephropathy, the loss of integrity of the glomerular filtration barrier occurs and leads to FSGS, which is one of the main findings in NS patients. FSGS is caused by loss of podocytes due to cell damage or apoptosis^33,34^ and is sometimes found in chronic kidney disease (CKD). Particularly, podocyte damage induces urinary protein leakage and detachment of podocyte from glomeruli followed by glomerulosclerosis^35^. In this study, we revealed that MES + HS ably suppressed proteinuria and glomerulosclerosis in ADR-induced FSGS mouse model. The potential of MES + HS to improve the pathologies in this mouse model is consistent with our previous study showing that MES + HS ameliorated the progressive proteinuria and inflammation in a mouse model of Alport syndrome, which is CKD caused by type 4 alpha collagen (COL4A) mutation^23^. In our current study, we showed that MES + HS inactivated the apoptotic factor BAD via the phosphorylation of Akt protein, resulting in the reduced number of TUNEL-positive cells and the decreased expression level of CC3 protein. This suggested that MES + HS has an anti-apoptotic effect. The cell types in which Akt signaling is activated and the subsequent apoptosis inhibition in vivo, however, remain to be determined. The involvement of Akt in the anti-apoptotic effect of MES + HS was evidenced by the treatment of MPC5 cells with Akt inhibitor LY294002, which abolished the effect of MES + HS-induced suppression of CC3 and phosphorylated BAD. The activation of Akt signaling by MES + HS had also been shown previously in our investigation on Alport syndrome^23^. The beneficial regulation of Akt-BAD signaling was demonstrated in a previous report in which Notch-2 agonistic monoclonal antibody ameliorated protein leakage and glomerulosclerosis in ADR-induced FSGS mice via regulating Akt-BAD axis^36^. Based on previous findings and the current study, activating Akt-BAD axis or inhibition of apoptosis has potential significance in controlling FSGS pathology.

Inflammation is a critical event in the progression phase and also an important determinant in the severity of FSGS. Immune cells infiltrate into kidney tissue during injury and produce proinflammatory cytokines such as *Tnfa* and *Il1b*. These cytokines promote epithelial mesenchymal transition and activates myofibroblasts to cause renal fibrosis^37^. Furthermore, *Tnfa* and *Il1b* accelerate fibrosis by increasing the expression level of *Il6* that promotes the self-proliferation of fibroblasts^29^. Hence, novel therapies targeting inflammation and fibrosis are being developed. In our study, we found that MES + HS reduced the expression levels of these pro-inflammatory cytokines in ADR-induced FSGS mice. In addition, we have reported that MES + HS suppressed the expressions of pro-inflammatory cytokines in Alport syndrome mice in in vivo and ex vivo experiments via regulation of JNK1/2 or p38 signaling^23^. Based on these results, we conclude that the anti-inflammatory effect of MES + HS also contributes to the renoprotective characteristic of MES + HS. Currently, steroids and immunosuppressants that have anti-inflammatory action are used for FSGS treatment^38–40^. Although these agents strongly suppress inflammation and exert therapeutic effect, some cases are steroid-resistant. In recent years, rituximab that recognizes CD20 expressed on the surface of B cells has been applied to FSGS treatment. It also shows efficacy against FSGS via suppression of inflammation or immune system, but its effects on steroid-resistant cases are controversial^39,41,42^. For refractory cases, it is hoped that new therapies will be developed. In this study, we found that MES + HS ameliorated renal pathology by suppressing cell damage through the Akt-BAD axis, which is different from the existing approaches. Because the mode of action between MES + HS and conventional agents is different, we consider that the combination therapy of these may be effective. In clinical studies, we have already optimized the treatment manner of MES + HS in humans. Furthermore, when a combination therapy of antidiabetic agent, dipeptidyl peptidase-4 (DPP4) inhibitor, and MES + HS was performed in patients with T2DM, additive or synergistic effects were observed^20^.

In conclusion, MES + HS ameliorated urinary protein leakage and glomerulosclerosis in a murine model of ADR nephropathy via regulation of Akt-BAD axis. Taking into consideration its anti-apoptotic and anti-inflammatory effects, MES + HS has renoprotective function that, together with its apparent safeness, may have good potential in controlling the pathology of FSGS.

## Materials and methods

### Animals

Male BALB/c mice were purchased from Charles River Laboratories Inc. (Kanagawa, Japan). To generate adriamycin-induced NS model, 8-week-old mice were injected with 10 mg/kg of adriamycin (Sigma-Aldrich Co. LLC., USA) via the tail vein^24^. Control mice were treated with an equivalent volume of normal saline vehicle. For urine collection, mice were placed in metabolic cages for 24 h at the indicated day in Fig. 1a. Within the duration of the experiment, mice were housed in a vivarium at the animal facility, and provided with food and water ad libitum. After 28 days from ADR injection, all mice were sacrificed and renal tissues were surgically removed for analysis. All the animal experiments were approved by the Animal Care and Use Committee of Kumamoto University (C28-068). All methods were performed in accordance with the relevant guidelines and regulations.

### MES + HS treatment on in vivo experiments

For MES + HS treatment, 8-week-old BALB/c mice were treated with MES + HS for 10 min twice a week within the duration of the experiment. Briefly, mice were placed in well-ventilated chamber that is attached with electro-conductive and thermo-generative rubber electrodes previously described in^17^. The condition of MES was 12 V direct current with individual pulse duration of 0.1 ms at 55 pulses per second (pps). Simultaneous HS treatment was applied at a temperature of 42 °C. These treatment conditions have been optimized and reported in our previous studies^17,19,23^. The mice in the control and ADR groups were placed in the above chamber for 10 min without MES + HS treatment.

### Quantitative analysis of albuminuria and protein/creatinine concentration

Urine samples were collected using metabolic cage within 24 h twice a week. Urinary volume collected within 24 h was measured. Urinary albumin concentration was assessed by 12% SDS-PAGE followed by CBB staining. Densitometric analysis was performed using ImageJ software and the data were normalized with BSA standard (0.5, 1, 5, 10 μg). The amount of leaked albumin was calculated by multiplying the measured concentration of albumin by urine volume to correct for differences in volume. Urinary protein was measured by Bradford assay (Bio-Rad) and creatinine was assessed by Jaffe’s method using creatinine kit (Wako). Urinary protein concentration was normalized with urinary creatinine concentration.

### Histological and immunohistochemical analysis

Mouse kidneys were collected at 28 days after ADR administration. Renal tissues were fixed in 10% formalin and embedded in paraffin for periodic acid Schiff (PAS) staining, immunohistochemical, and TUNEL staining. Tissue blocks were sliced into 4-μm thickness using microtome. For PAS staining, paraffin sections were stained by PAS staining kit (Sigma-Aldrich Co. LLC., USA) following the recommended protocol. To evaluate the glomerulosclerosis severity, more than 50 random glomeruli per mouse were scored based on the following criteria as reported previously^43^, 0: no lesion, 1: expansion of mesangial area, 2: expansion of Bowman’s epithelial cells, adhesion of glomeruli and Bowman’s capsule and partial sclerosis, 3: sclerotic area in 50–75% of glomeruli, and 4: sclerotic area in 75–100% of glomeruli. In addition, 10 random fields per mouse were captured and protein cast area (%) was calculated by the ratio to tissue area. For immunohistochemical staining, antigen retrieval was performed with 1 mM EDTA pH 8.0 at 121 °C for 20 min. After quenching of endogenous peroxidase with 0.3% H_2_O_2_ in methanol at room temperature for 15 min, sections were incubated sequentially for 15 min each with Avidin solution and Biotin solution (Avidin/Biotin Blocking Kit; Vector Laboratories, USA). Then, incubated sections were blocked with Protein Block Serum-Free (Dako, USA) at room temperature for 30 min and reacted with primary antibody diluted at 1:500 with Antibody Diluent with Background Reducing Components (Dako, USA). The next day, sections were incubated at room temperature for 1 h with secondary antibody diluted at 1:1000 prior to application of VECTASTAIN Elite ABC Reagent (Vector Laboratories, USA). Polyclonal rabbit anti-WT-1 (sc-192; Santa Cruz Biotechnology) and Goat anti-rabbit IgG antibody (H + L)-biotinylated (BA-1000; Vector Laboratories) were used for antibody reaction. The bound antibody was visualized using 3,3′-Diaminobenzidine (DAB). WT-1-positive cells in 50 random glomeruli per mouse were counted and expressed as average per glomerulus. For TdT-mediated dUTP Nick-End Labeling (TUNEL) staining, paraffin sections were stained by Dead End Fluorometric TUNEL System (Promega, USA) following the manufacturer’s recommendation. Sections were counterstained with PI solution (1 μg/mL). Random 10 fields per mouse were captured and apoptosis rate was calculated by the ratio of TUNEL-positive cells to PI-positive cells. All images were acquired using BIOREVO microscope and analysis software (Keyence, Japan).

### Immunofluorescence

Mouse kidneys were collected at 28 days after ADR administration. Renal tissues were frozen with liquid nitrogen in OCT compound immediately after collection. Frozen tissues were sliced into 10-μm thickness using cryostat and attached onto glass slides. Sections were thawed with ice-cold acetone at − 20 °C for 5 min. After blocking with Protein Block Serum-Free (Dako, USA) at room temperature for 30 min, sections were reacted with primary antibody diluted at 1:100 and secondary antibody diluted at 1:300 with Antibody Diluent with Background Reducing Components (Dako, USA). Primary antibody was reacted overnight at 4 °C and secondary antibody was reacted for 1 h at room temperature. Polyclonal rabbit anti-WT-1 (sc-192; Santa Cruz Biotechnology), purified rat anti-mouse CD44 (#550538; BD, USA), Goat anti-rabbit IgG (H + L) Alexa Fluor 488 (A-11008; Thermo Scientific Inc., USA) and Goat anti-rat IgG (H + L) Alexa Fluor 488 (A-11006; Thermo scientific Inc., USA) were used for antibody reactions. Sections were incubated with 1 μg/mL of 4′,6-diamidino-2-phenylindole (DAPI) solution for 20 min at room temperature. Then, stained sections were mounted with VECTASHIELD mounting medium (Vector Laboratories, USA). Images were captured using BIOREVO microscope (Keyence, Japan).

### Immunoblotting analysis

To determine the protein expression level, whole kidney samples and MPC5 cells were lysed in lysis buffer (25 nmol/mL HEPES, 10 mmol/mL Na_4_P_2_O_7_∙10H_2_O, 100 mmol/mL NaF, 5 mmol/mL EDTA, 2 mmol/mL Na_3_VO_4_, 1% Triton X-100) and protein lysates were subjected to SDS-PAGE and Western blot analysis as described previously^19^. Anti-phospho-Akt (#9271), anti-Akt (#9272), anti-phospho-BAD (#4366), anti-BAD (#9239) and anti-Cleaved caspase 3 (#9661) antibodies from Cell Signaling Technology were used for antibody reaction. Anti-actin (sc-1616; Santa Cruz Biotechnology) antibody was used as loading control. The above primary antibodies were detected using their respective HRP-conjugated secondary antibodies. SuperSignal West Pico PLUS Chemiluminescent Substrate (Thermo Scientific Inc., USA) and Amersham ECL Prime Western Blotting Detection Reagent (GE Healthcare Life Science, USA) were used for visualizing the blots.

### Real-time quantitative RT-PCR analysis (Q-PCR)

Total RNA was isolated from mice renal tissues using RNAiso Plus (Takara, Japan) with homogenization. Reverse transcription and PCR amplification were performed using PrimeScript RT Reagent Kit and SYBR Premix ExTaq II (Takara, Japan), respectively, as described in^44^. The sequences of primers used for Q-PCR are listed in Table 1.

**Table 1:** Real-time quantitative RT-PCR mouse primers.

Gene	Sense	Antisense
*Lipocalin2*	5′-GAGAAGGCAGCTTTACGATG-3′	5′-CCTGGAGCTTGGAACAAATG-3′
*Lysozyme*	5′-CCAGTGTCACGAGGCATTCA-3′	5′-TGATAACAGGCTCATCTGTCTCA-3′
*Tnfa*	5′-CATCTTCTCAAAATTCGAGTGACAA-3′	5′-TGGGAGTAGACAAGGTACAACCC-3′
*Il1b*	5′-GCTGAAAGCTCTCCACCTCAATG-3′	5′-TGTCGTTGCTTGGTTCT CCTTG-3′
*Il6*	5′-GAGGATACCACTCCCAACAGACC-3′	5′-AAGTGCATCATCGTTGTTCATACA-3′
*Kc/Il8*	5′-TGTCAGTGCCTGCAGACCAT-3′	5′-GAGCCTTAGTTTGGACAGGATCTG-3′
*Tgfb*	5′-CACCTGCAAGACCATCGACAT-3′	5′-GAGCCTTAGTTTGGACAGGATCTG-3′
*aSma*	5′-CCCAGACATCAGGGAGTAATGG-3′	5′-TCTATCGGATACTTCAGCGTCA-3′
*Col1a1*	5′-CTGGCGGTTCAGGTCCAAT-3′	5′-TTCCAGGCAATCCACGAGC-3′
*18S rRNA*	5′-GTAACCCGTTGAACCCCATT-3′	5′-CCATCCAATCGGTAGTAGCG-3′

### Cell culture and in vitro MES + HS treatment

The conditionally immortalized mouse podocyte clone 5 (MPC5) was a kind gift from Dr. Peter Mundel^45^. Non-differentiated MPC5 cells were cultured at 33 °C and maintained in RPMI-1640 containing 10% fetal bovine serum (FBS), 100 units/mL penicillin and 100 μg/mL streptomycin, and 10 units/mL recombinant mouse IFN-γ. To differentiate MPC5, cells were cultured at 37 °C for 10–14 days without IFN-γ. After differentiation, MPC5 cells were incubated with 200 nmol/mL of adriamycin (ADR, Sigma-Aldrich Co. LLC, USA) and treated with MES + HS. MES + HS treatment was performed as described previously^17,19,23^. Briefly, MPC5 cells were plated on 35-mm culture dishes and treated with MES + HS for 10 min. For MES treatment, the cover of culture plate was exchanged with a plate cover attached with carbon electrodes (IonOptix) and connected to a function generator (WF1973; NF Corporation, Japan). The function generator was set to produce 1 V/cm direct current with individual pulse duration of 0.1 ms and pulse rate of 55 pps^46^. HS treatment was delivered simultaneously by putting the sealed culture plate with electrodes in water bath at a temperature of 42 °C. After MES + HS treatment, the media were changed, and cells were incubated at 37 °C until assay.

### Flow cytometry

Apoptotic cells were detected using DeadEnd Fluorometric TUNEL System (Promega, USA) following the recommended protocol. Briefly, harvested cells (3–5 × 10^5^ cells/mL) were washed twice with PBS and fixed with 1% paraformaldehyde. After permeabilization by Triton X-100, fragmented DNA was labeled with fluorescein. Stained cells were analyzed using BD Accuri C6 (BD Bioscience, USA). Data were analyzed using Flowlogic software (Miltenyi Biotec, USA).

### Lactate dehydrogenase (LDH) assay

To analyze for LDH release, treated MPC5 cells were incubated for 24 h. Then culture media were centrifuged at 12,000 rpm for 15 min, and cleared supernatants were collected. Cell pellets and the remaining attached cells in the plates were lysed with 1% Triton X-100 solution. Media and lysates were subjected to LDH assay using cytotoxicity detection kit (Roche Applied Science, Germany) following the recommended protocol. LDH release was calculated by the ratio of LDH concentration in the media to total LDH concentration (media and lysate).

### Statistical analysis

All data are presented as mean ± SE. Significance of the difference between groups were assessed by ANOVA with Tukey–Kramer test. *P* value less than 0.05 was considered as statistically significant.

## Supplementary information

Supplementary Figures.

## Data Availability

All data generated or analyzed during this study are included in this published article and its Supplementary Information files.
