# Supplementary material for: Mild electrical stimulation with heat shock attenuates renal pathology in adriamycin-induced nephrotic syndrome mouse model
Source: Sci Rep. 2020 Oct 30;10:18719. doi: 10.1038/s41598-020-75761-8 (PMC7603347; doi:10.1038/s41598-020-75761-8)
Supplement: Supplementary file 1 — Supplementary Figures. [file 41598_2020_75761_MOESM1_ESM.pdf]

Supplementary Information

Mild electrical stimulation with heat shock attenuates renal pathology in adriamycin-induced nephrotic syndrome mouse model

Keisuke Teramoto[1,2], Yu Tsurekawa[1,2], Mary Ann Suico[1,3], Shota Kaseda[1,2], Kohei Omachi[1,2], Tsubasa Yokota[1], Misato Kamura[1,2], Mariam Piruzyan[1,2], Tatsuya Kondo[4], Tsuyoshi Shuto[1,3], Eiichi Araki[4], Hirofumi Kai[1,2,3]*

[1]Department of Molecular Medicine, Graduate School of Pharmaceutical Sciences, Kumamoto University, Kumamoto, Japan. [2]Program for Leading Graduate Schools "HIGO (Health life science: Interdisciplinary and Global Oriented) Program", Kumamoto University, Kumamoto, Japan. [3]Global Center for Natural Resources Sciences, Faculty of Life Sciences, Kumamoto University, 5-1 Oe-Honmachi, Chuo-ku, Kumamoto City 862-0973, Kumamoto, Japan. [4]Department of Metabolic Medicine, Faculty of Life Sciences, Kumamoto University, 1-1-1 Honjo, Chuo-ku, Kumamoto City 860-8556, Kumamoto, Japan.

Supplementary Figure 1.

Fig. 4c

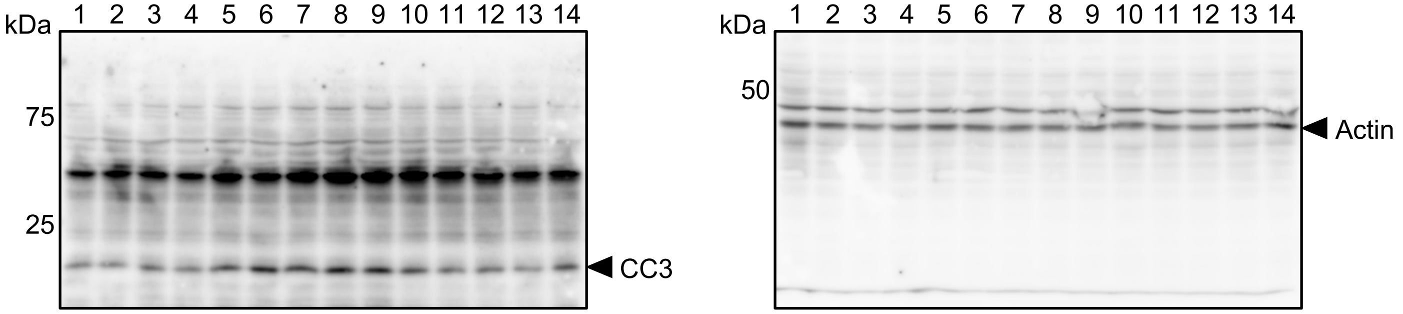

Fig. 4d

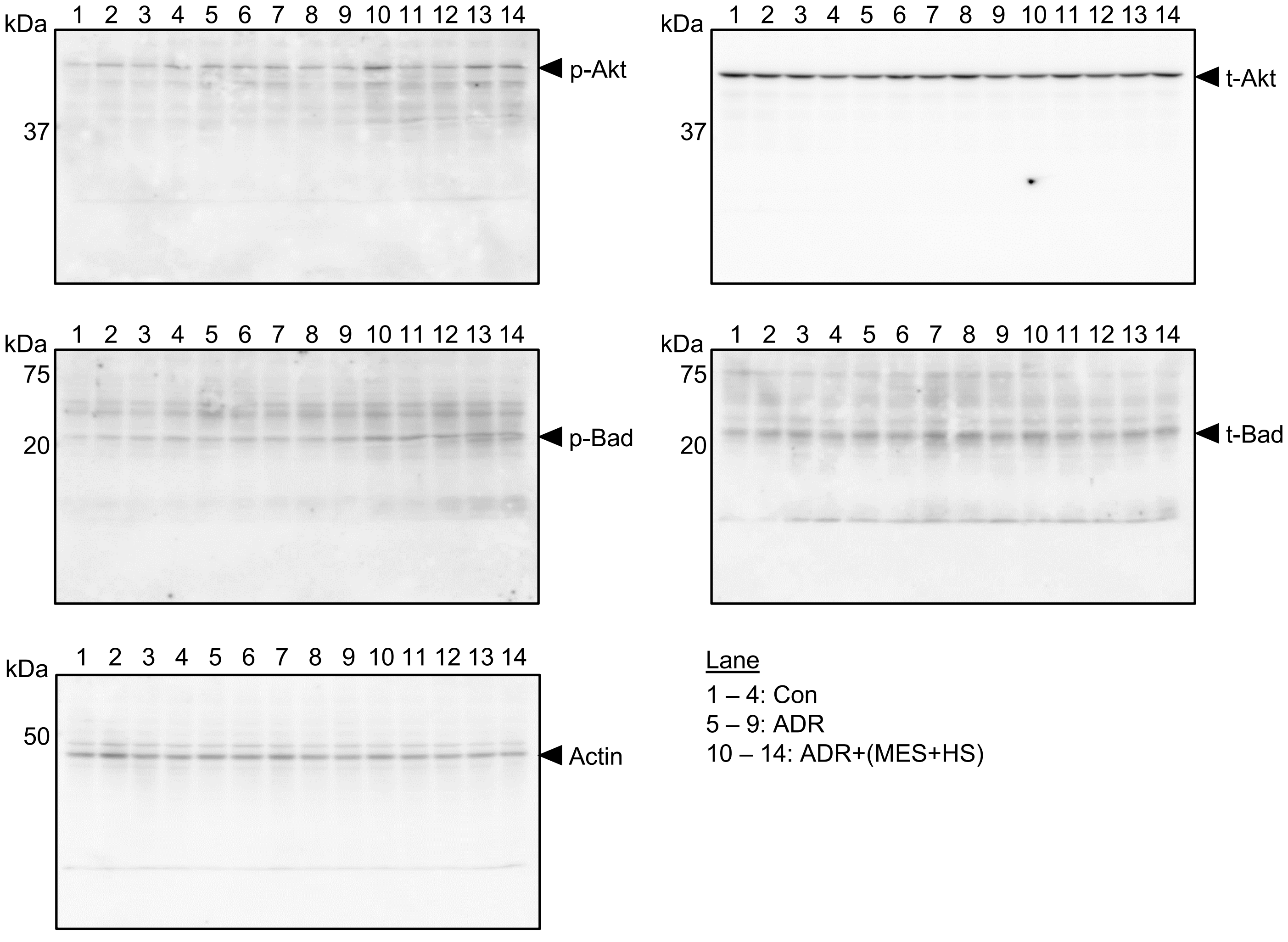

Supplementary Figure 1. The full-length blots for Figure 4c and 4d.
The full-length blots for Figure 4c and d with the indicated antibodies. Actin was used as loading control. Samples were derived from the same experiment, and gels/blots were processed in parallel.

Supplementary Figure 2.

Fig. 5d

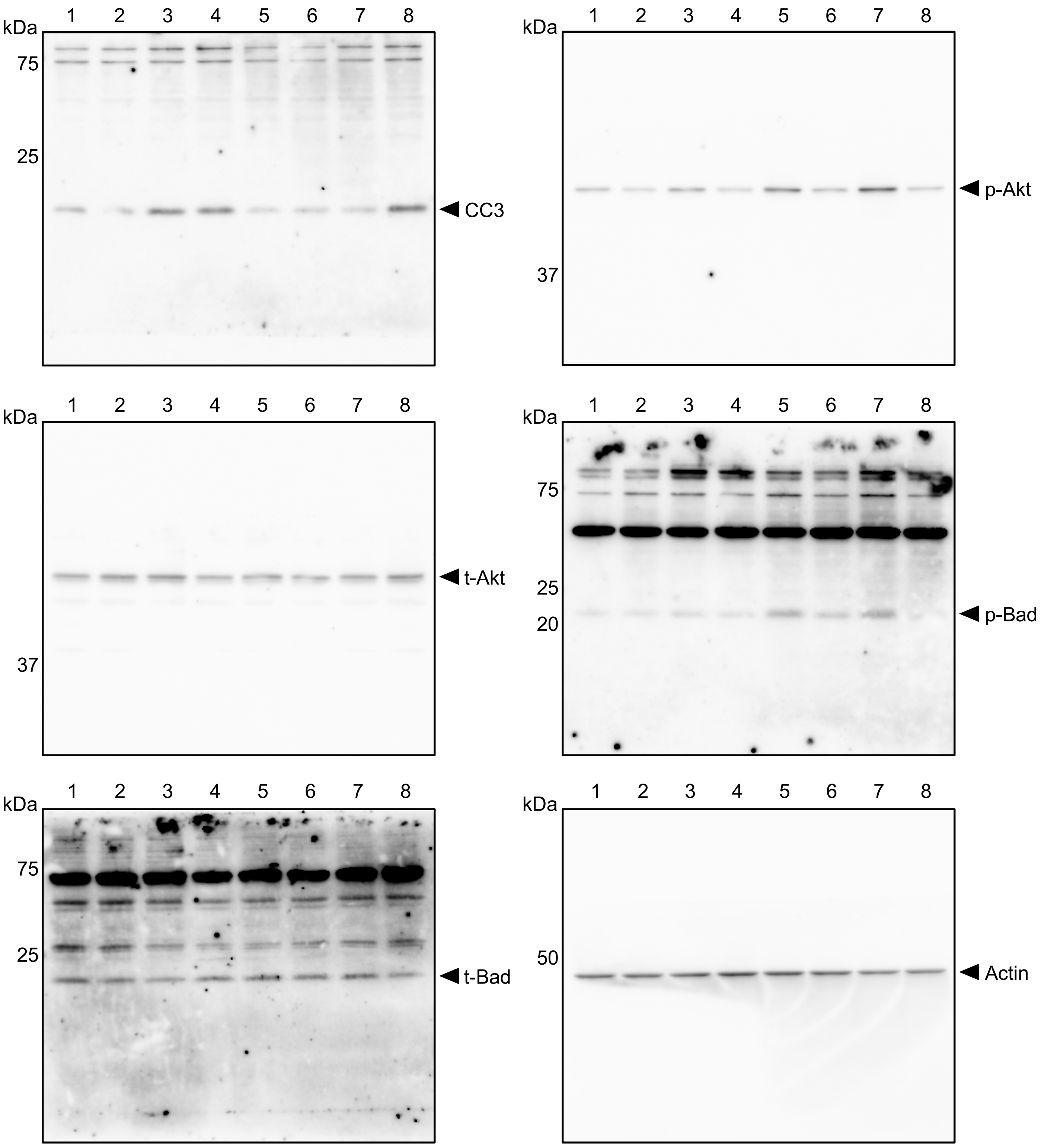

Lane

1: Con
2: LY294002
3: ADR
4: LY294002 + ADR
5: MES+HS
6: LY294002 + (MES+HS)
7: ADR + (MES+HS)
8: LY294002 + ADR + (MES+HS)

Supplementary Figure 2. The full-length blots for Figure 5d.
The full-length blots for Figure 5d with the indicated antibodies. Actin was used as loading control. Samples were derived from the same experiment, and gels/blots were processed in parallel.
